# Phosphatidylserine synthase 2 and phosphatidylserine decarboxylase are essential for aminophospholipid synthesis in *T* *rypanosoma brucei*

**DOI:** 10.1111/mmi.13637

**Published:** 2017-03-02

**Authors:** Luce Farine, Jennifer Jelk, Jae‐Yeon Choi, Dennis R. Voelker, Jon Nunes, Terry K. Smith, Peter Bütikofer

**Affiliations:** ^1^ Institute of Biochemistry and Molecular MedicineUniversity of BernBern3012Switzerland; ^2^ Department of MedicineNational Jewish HealthDenverCO80206USA; ^3^ Biomedical Sciences Research ComplexUniversity of St. AndrewsSt. AndrewsScotland

## Abstract

Phosphatidylethanolamine (PE) and phosphatidylserine (PS) are ubiquitously expressed and metabolically interconnected glycerophospholipids in eukaryotes and prokaryotes. In *Trypanosoma brucei*, PE synthesis has been shown to occur mainly via the Kennedy pathway, one of the three routes leading to PE synthesis in eukaryotes, while PS synthesis has not been studied experimentally. We now reveal the importance of *T. brucei* PS synthase 2 (TbPSS2) and *T. brucei* PS decarboxylase (TbPSD), two key enzymes involved in aminophospholipid synthesis, for trypanosome viability. By using tetracycline‐inducible down‐regulation of gene expression and *in vivo* and *in vitro* metabolic labeling, we found that TbPSS2 (i) is necessary for normal growth of procyclic trypanosomes, (ii) localizes to the endoplasmic reticulum and (iii) represents the unique route for PS formation in *T. brucei*. In addition, we identified TbPSD as type I PS decarboxylase in the mitochondrion and found that it is processed proteolytically at a WGSS cleavage site into a heterodimer. Down‐regulation of TbPSD expression affected mitochondrial integrity in both procyclic and bloodstream form trypanosomes, decreased ATP production via oxidative phosphorylation in procyclic form and affected parasite growth.

## Introduction

Glycerophospholipids are major building blocks of all biological membranes. The relative abundance of the different classes and their subclass and molecular species compositions not only modulate membrane characteristics, such as fluidity, curvature and membrane tension, but also affect properties of membrane‐associated proteins (reviewed by Lee, 2004). The aminophospholipid classes, phosphatidylethanolamine (PE) and phosphatidylserine (PS), are present in membranes of most eukaryotes and prokaryotes (reviewed by Vance, 2008; Vance and Tasseva, 2013). PE has been shown to be involved in a wide range of biological processes, including cell division (Emoto *et al*., 1996) and protein folding (Bogdanov and Dowhan, 1998), and represents the ethanolamine donor for the synthesis of glycosylphosphatidylinositol (GPI) anchors (Menon and Stevens, 1992) and other protein modifications (Ichimura *et al*., 2000; Signorell *et al.*, 2008a; Cullen and Trent, 2010). PS functions as an important precursor for some pools of PE (reviewed by Vance and Steenbergen, 2005), acts as a cofactor for enzymes involved in signaling pathways (Takai *et al*., 1979), and its exposure at the cell surface is an early event in apoptosis (Fadok *et al*., 1992) and serves as a critical cofactor in blood clotting (Bevers *et al*., 1983). The pathways for the synthesis of PS and PE are often coupled, but the contributions of the individual pathways and their interconnections differ considerably among eukaryotes (reviewed by Vance and Tasseva, 2013).

Decarboxylation of PS represents a main route for PE synthesis in many organisms (Borkenhagen *et al*., 1961; reviewed by Schuiki and Daum, 2009). It is the major pathway for PE production in bacteria and the major route in *Saccharomyces cerevisiae*, however it is also active in mammals and plants. In addition, in mammalian cells, plants and yeast, PE can be formed via the CDP‐ethanolamine branch of the Kennedy pathway (Kennedy and Weiss, 1956; reviewed by Vance, 2008; Gibellini and Smith, 2010). A third pathway for PE synthesis in many eukaryotes involves head group exchange with PS (Suzuki and Kanfer, 1985; reviewed by Vance, 2008). PS decarboxylases (PSDs) in prokaryotes and eukaryotes consist of two different types. Type I PSDs are present in eukaryotic mitochondria and bacteria whereas type II PSDs comprise eukaryotic enzymes located in the endomembrane system (Golgi, endoplasmic reticulum (ER), vacuole/tonoplast) (reviewed by Schuiki and Daum, 2009). PSDs are usually transmembrane proteins that are active as heterodimers composed of an α‐ and a β‐subunit. The subunits are generated from a proenzyme via autocatalytic cleavage at a conserved recognition motif close to the C‐terminus (GSS/T), generating a long N‐terminal (transmembrane) β‐subunit and a short C‐terminal α‐subunit (Trotter *et al*., 1995; Kitamura *et al*., 2002). PSDs contain an unusual pyruvoyl prosthetic group, which is generated at the amino terminus of the α‐subunit from the serine residue located at the cleavage site of the proenzyme. Although the two PSD subunits remain tightly associated after the cleavage, they are not covalently linked (reviewed by van Poelje and Snell, 1990; Schuiki and Daum, 2009).

Bacteria and mammalian cells contain a single type I PSD, while *S. cerevisiae* express a mitochondrial type I PSD (responsible for 80% of PSD activity) and a type II PSD localized in the Golgi (reviewed by Vance and Steenbergen, 2005; Schuiki and Daum, 2009). Yeast mutants lacking both PSD enzymes are auxotrophic for ethanolamine, which is used for PE synthesis via the CDP‐ethanolamine pathway (Trotter and Voelker, 1995). Plants contain a mitochondrial *psd1* and two type II PSDs localized in the endomembrane system (Nerlich *et al*., 2007). Interestingly, unique forms of PSD have been identified in protozoan parasites. An ER‐localized type I PSD has been described in *Plasmodium falciparum* (Baunaure *et al*., 2003), representing the only known non‐mitochondrial type I PSD so far, whereas a soluble PSD secreted into the parasitophorus vacuole has been described in *Toxoplasma gondii* (Gupta *et al*., 2012).

PS can be synthesized by two different pathways. PS synthase (PSS) uses CDP‐diacylglycerol (CDP‐DAG) and serine as substrates, while PS synthase 1 (PSS1) and PS synthase 2 (PSS2) generate PS via calcium‐dependent head group exchange reactions using PC and PE, respectively, as substrates. PSS enzymes are membrane‐bound and have been detected in membrane fractions of bacteria (Matsumoto, 1997) and in the outer mitochondrial and ribosomal fraction of yeast cells (Yamashita and Nikawa, 1997). Depletion of PSS causes a conditional lethal temperature‐sensitive growth phenotype in *Escherichia coli* (Ohta and Shibuya, 1977) and renders yeast auxotrophic for ethanolamine/choline (Nikawa and Yamashita, 1981). In contrast, mammalian PSS1/2 enzymes have been localized to mitochondria‐associated membranes (MAMs) (Stone and Vance, 2000). MAMs represent a particular membrane fraction with distinct biochemical properties harboring proteins that are involved in the synthesis and transport of lipids and mediate import of PS into mitochondria for decarboxylation to PE (reviewed by Vance, 2014). Comparative schemes with biosynthetic connections between PC, PE, and PS in eukaryotes are shown in Supporting Information Fig. S1.

The pathways for *de novo* synthesis of PE have also been studied in the very early‐branching eukaryote *Trypanosoma brucei* (reviewed by Serricchio and Bütikofer, 2011; Farine and Bütikofer, 2013). *T. brucei* is the causative agent of human African trypanosomiasis, also called sleeping sickness, and nagana a related livestock disease. The diseases are fatal unless treated and represent a major cause of poverty in sub‐Saharan Africa (Fevre *et al*., 2008). Trypanosomes alternate between an insect vector, the tsetse, where they replicate as procyclic forms in the midgut and as epimastigotes in the salivary glands, and a mammalian host, where they replicate as long slender bloodstream forms (Vickerman, 1985).

It has been shown previously that the CDP‐ethanolamine branch of the Kennedy pathway is essential for growth of *T. brucei* procyclic and bloodstream forms in culture (Gibellini *et al*., 2008; Signorell *et al.*, 2008b; Gibellini *et al*., 2009; Signorell *et al*., 2009). In addition, experimental data in procyclic forms (Signorell *et al.*, 2008b) together with bioinformatic analysis of the *T. brucei* genome suggested that two other possible routes may contribute to PE formation in *T. brucei*: decarboxylation of PS by putative TbPSD (Tb927.9.10080) and head group exchange with PS by putative TbPSS2 (Tb927.7.3760). However, the respective enzymes have not been characterized experimentally and their essentiality has not been investigated. In the present study, we characterize TbPSD as mitochondrial type I PSD. Its down‐regulation affects cell growth, mitochondrial integrity and ATP production by oxidative phosphorylation. In addition, we demonstrate that base exchange between PE and PS is catalyzed by TbPSS2 and represents the only pathway for PS production in *T. brucei*. Expression of TbPSS2 is essential for normal growth of *T. brucei* in culture.

## Results

### Tb927.7.3760 encodes a PS synthase 2

*In silico* analysis of putative TbPSS2 (Tb927.7.3760) protein sequence revealed the presence of 8 to 9 membrane spanning regions (Phobius, TriTrypDB) and a domain between amino acids 135 to 412 belonging to the PSS pfam family (PF03034). The PSS family comprises base exchange enzymes that replace the existing head group of a phospholipid with l‐serine, such as mammalian PSS1 and PSS2 (Finn *et al*., 2014). PSS enzymes are mechanistically not related to PS synthases from yeast (CHO1), which are members of the CDP‐alcohol phosphatidyltransferase protein family. On the amino acid level, bioinformatics prediction tools (Pairwise Sequence Alignment, EMBOSS, EMBL‐EBI) revealed 23.7% and 26.2% identity (37.1% and 39.4% similarity) between TbPSS2 and human PSS1 (isoform 1) and PSS2, respectively, but only 11.8% identity (19.8% similarity) between TbPSS2 and *S. cerevisiae* PSS, which is consistent with TbPSS2 representing a base exchange enzyme. This was confirmed by over‐expressing TbPSS2 in *E. coli* (Supporting Information Fig. S2A) and assaying a membrane preparation for PSS activity in the presence of various substrates (Supporting Information Fig. S2B). Low PSS activity was observed in the presence of CDP‐DAG as substrate, likely due to the action of endogenous *E. coli* PSS. In contrast, in the presence of PE, strong head group exchange activity with [^3^H]serine was observed in TbPSS2‐overexpressing membranes compared to control membranes.

### Down‐regulation of TbPSS2 inhibits growth of procyclic form trypanosomes

**Figure 1:**
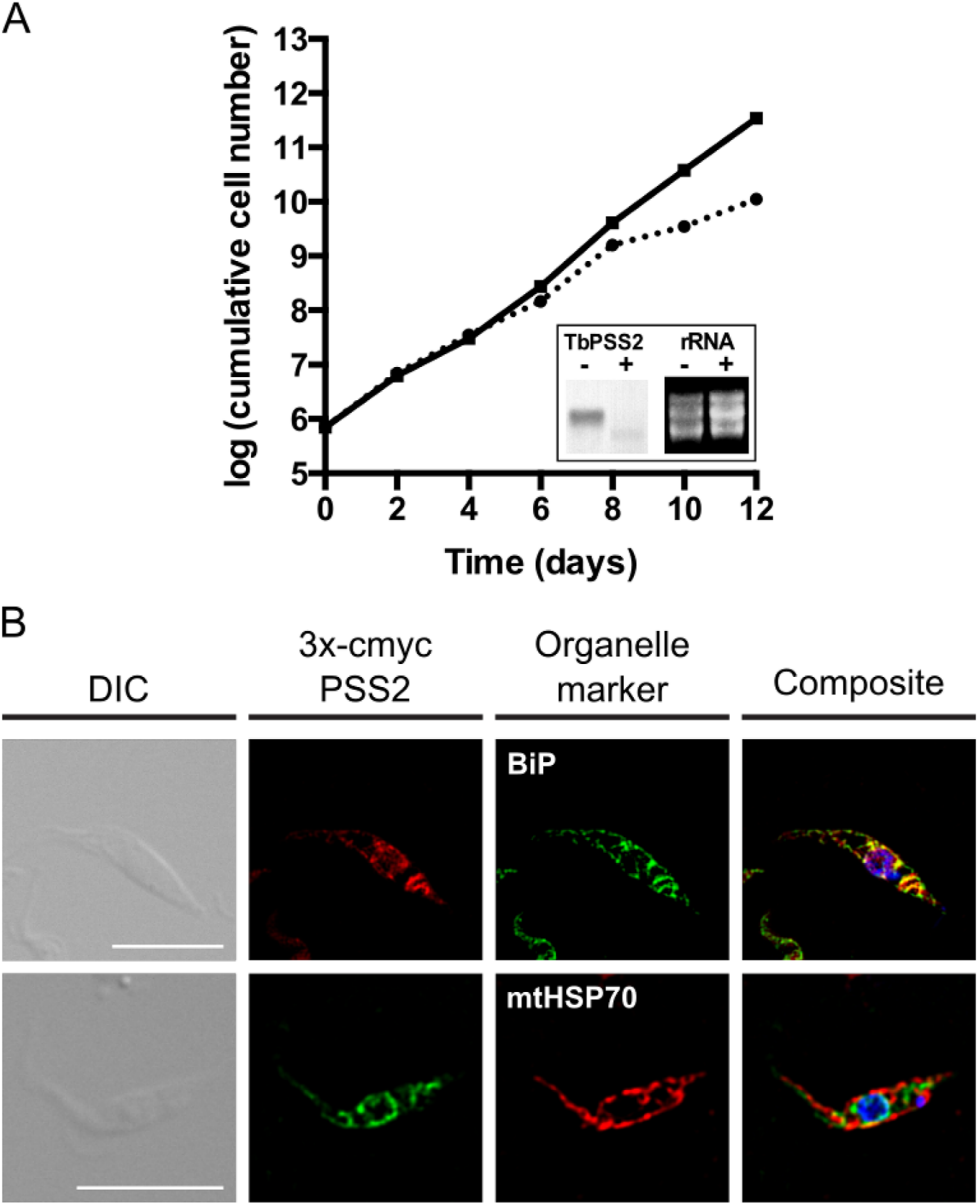
Growth of TbPSS2‐depleted parasites and localization of TbPSS2. A. Growth of control (solid line) and TbPSS2‐depleted (dotted line) procyclic form trypanosomes. The data points represent mean values from single measurements using two different clones. The inset shows a Northern blot analysis of TbPSS2 mRNA levels (left two lanes) in parasites incubated in the absence (−) or presence (+) of tetracycline for 2 days. rRNA levels are shown as loading controls (right two lanes). B. Subcellular localization of TbPSS2 in procyclic forms. Trypanosomes expressing 3xc‐myc‐tagged TbPSS2 were immunostained with anti‐c‐myc antibody. BiP and mtHSP70 were used as endoplasmic reticulum and mitochondrial marker, respectively. Scale bars indicate 10 µm.

Essentiality of TbPSS2 for parasite growth was assessed using tetracycline‐inducible RNAi‐mediated down‐regulation of TbPSS2. After 3 days of tetracycline induction, TbPSS2 mRNA levels showed efficient down‐regulation (Fig. 1A) and parasite growth in the presence of tetracycline was reduced compared to uninduced cells after 6 days of culture, demonstrating that expression of TbPSS2 is essential for normal growth of *T. brucei* procyclic forms in culture.

### TbPSS2 co‐localizes with the ER marker BiP

Localization of TbPSS2 in *T. brucei* procyclic forms was studied by expressing an inducible N‐terminally 3xc‐myc‐tagged form of TbPSS2. Immunofluorescence microscopy revealed good co‐localization of 3xc‐myc‐TbPSS2 with the ER marker BiP, whereas no co‐localization was observed with mitochondrial mtHSP70 (Fig. 1B).

### TbPSS2 is the only route for PS synthesis in *T. brucei*

**Figure 2:**
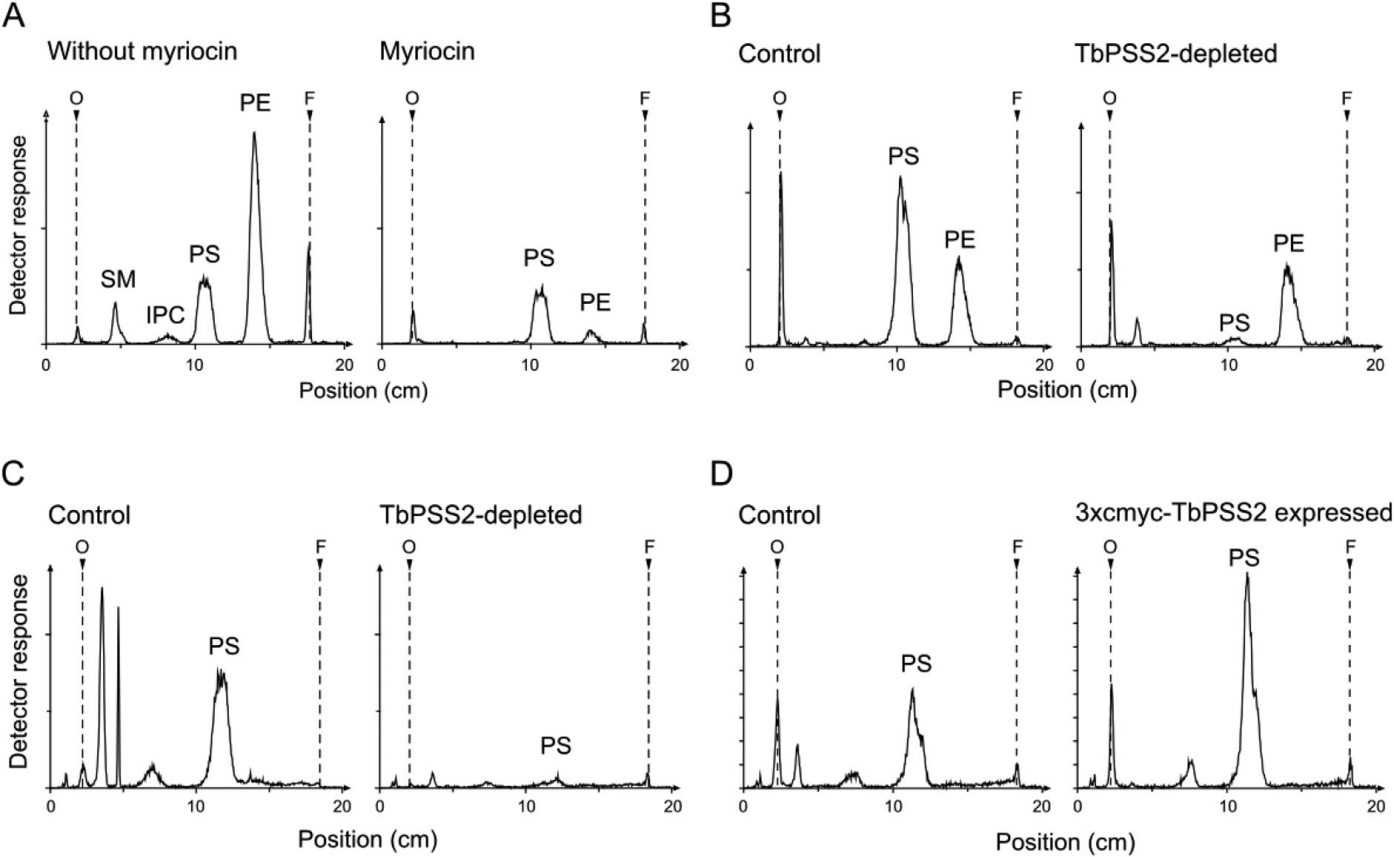
Lipid analysis of TbPSS2 parasites after [^3^H]serine labeling. Procyclic form trypanosomes or digitonin extracts were labeled with [^3^H]serine for 4 h and lipids were extracted, separated by one‐dimensional thin layer chromatography and detected using a radioisotope detector. A. [^3^H]Serine labeling of phospholipids in parasites before (left panel) and after 30 min of myriocin treatment (2.5 µM; right panel). B, C. [^3^H]Serine labeling of whole cells (panel B) and digitonin extracts (panel C) before and after RNAi‐mediated depletion of TbPSS2 for 6 days. D. [^3^H]Serine labeling of control and 3xc‐myc‐TbPSS2 overexpressing procyclic forms after 2 days of tetracycline induction. The scans are representative of at least three independent experiments.

To study the importance of TbPSS2 on PS synthesis in procyclic forms, RNAi‐suppressed parasites were labeled with [^3^H]serine and lipids were analyzed by one‐ and two‐dimensional thin layer chromatography (TLC) in combination with radioisotope scanning, fluorography and lipid phosphorus determination. Incubation of trypanosomes with [^3^H]serine is expected to not only label PS and PE (via decarboxylation of [^3^H]PS), but also the major sphingolipids, inositol phosphorylceramide (IPC) and sphingomyelin (SM), via serine palmitoyltransferase (Signorell *et al.*, 2008b). Since degradation of [^3^H]serine‐labeled sphingolipids via sphingosine‐1‐phosphate lyase may result in the formation of [^3^H]ethanolamine‐phosphate, which is prevalent in *Leishmania* (Zhang *et al*., 2007), which in turn can be used for [^3^H]PE synthesis via the Kennedy pathway, an inhibitor of serine palmitoyltransferase, myriocin, was used to block *de novo* synthesis of sphingolipids. As shown in Fig. 2A, and consistent with the *Leishmania* results (Zhang *et al*., 2007), the addition of myriocin completely blocked *de novo* formation of IPC and SM in *T. brucei* procyclic forms in culture, resulting in labeling of PS and PE only. These conditions were subsequently used to study [^3^H]serine‐labeling in parasites after down‐regulation of TbPSS2. The results show that RNAi against TbPSS2 for 3 days inhibited incorporation of [^3^H]serine into newly synthesized PS by 94.8 ± 1.0% (mean value ± standard deviation from three independent experiments) in intact parasites (Fig. 2B) and by 91.3 ± 1.0% (mean value ± standard deviation from three independent experiments) in digitonin extracts from TbPSS2‐depleted parasites (Fig. 2C). Interestingly, [^3^H]PE formation was not affected by down‐regulation of TbPSS2 (Fig. 2B) (remaining at 92.5 ± 14.6% of control levels; mean value ± standard deviation from three independent experiments) and no labeling of PE was observed in digitonin extracts from control and TbPSS2‐depleted cells (Fig. 2C). In addition, quantification by two‐dimensional TLC and lipid phosphorus determination of total phospholipids after down‐regulation of TbPSS2 for 4 days revealed a reduction in the steady‐state levels of PS from 3.0 ± 1.1% of total phospholipids in control parasites to 0.9 ± 0.2% in TbPSS2‐depleted cells (mean values ± standard deviations from four independent experiments), indicating decreased activity of TbPSS2 resulting in lower PS levels. This finding is consistent with the observation that PS could no longer be labeled with [^3^H]serine *in vivo* in trypanosomes after down‐regulation of TbPSS2 (Supporting Information Fig. S3). Together, these results identify TbPSS2 as the only route for *de novo* PS synthesis in *T. brucei*.

In addition, to study whether the N‐terminally tagged TbPSS2 used for sub‐cellular localization (see Fig. 1B) is functional, digitonin extracts from parasites expressing (inducible) 3xc‐myc‐TbPSS2 were assayed for enzyme activity. The results show increased (i.e. 2.2 ± 0.3‐fold; mean value ± standard deviation from three independent experiments) [^3^H]PS formation in extracts from TbPSS2 over‐expressing parasites compared to extracts from the same cell line before induction of 3xc‐myc‐TbPSS2 expression (Fig. 2D), indicating that tagged TbPSS2 is catalytically active.

### Tb927.9.10080 encodes a type I PS decarboxylase

**Figure 3:**
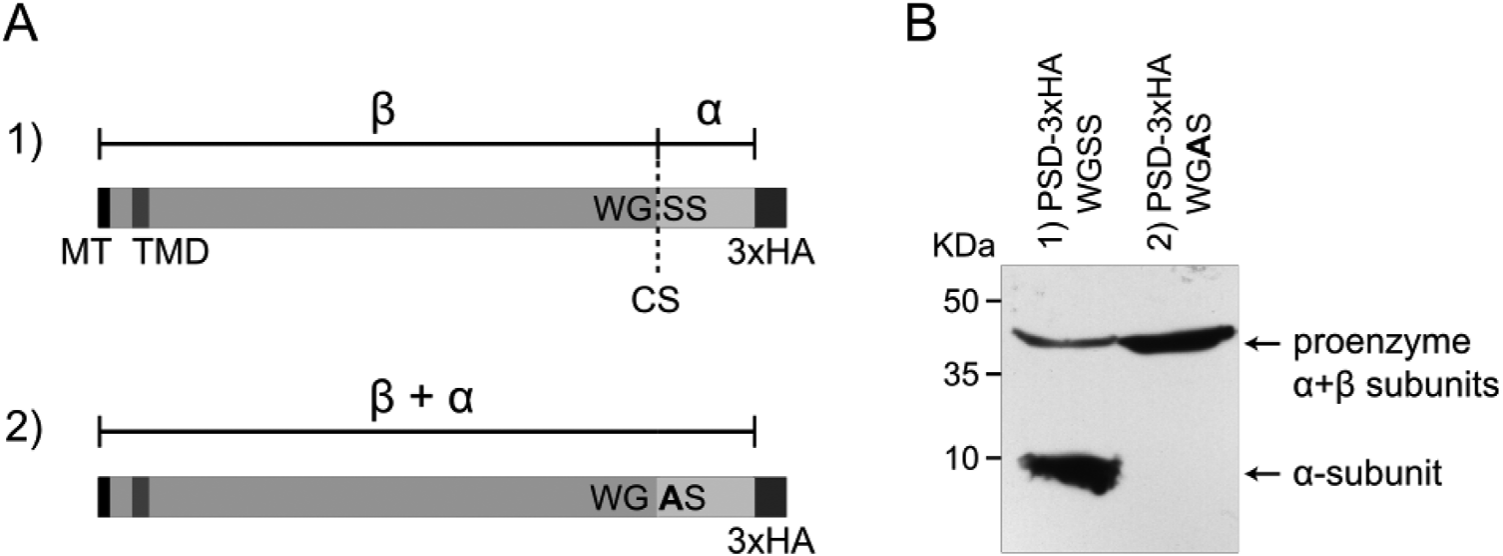
Processing of TbPSD. A. Predicted processing of wild‐type (1) and mutated (2) TbPSD into α‐ and β‐subunits. MT, mitochondrial targeting signal; TMD, transmembrane domain; CS, cleavage site; WGSS/WGAS, amino acid motifs at predicted cleavage site; 3xHA, C‐terminal attachment of 3xHA tag. B. SDS‐PAGE/immunoblot showing two bands representing the proenzyme (43.2 kDa) and the cleaved α‐subunit (7.4 kDa) (left lane), and a single band representing the uncleaved proenzyme after mutation of the recognition site (right lane).

Using prediction programs and amino acid sequence comparison tools, we identified Tb927.9.10080 as a candidate gene encoding putative *T. brucei* type I PSD (TbPSD). The sequence revealed a predicted mitochondrial targeting sequence (iPSORT prediction; Bannai *et al*., 2002) and a single transmembrane domain in the N‐terminal part of the protein (SMART; Schultz *et al*., 1998). The pfam domain PS Decarboxylase (PF02666) was located between amino acids 125 and 348 (Finn *et al*., 2014). Amino acid sequence alignments of TbPSD with *E. coli*, *P. falciparum, T. gondii, Arabidopsis thaliana* and human PSDs showed between 19.2 and 24.6% identity and revealed the presence of a putative proteolytic PSD cleavage recognition motif, WGSS (amino acids 316‐319) (Fig. 3A). The same sequence motif is also present in putative PSD homologs of other kinetoplastids, including *Leishmania major* (LmjF.35.4590) and *Crithidia fasciculata* (CFAC1.300097400). The predicted proteolytic cleavage motif of TbPSD is identical to that of *P. falciparum* PSD, rather than the typical GST motif of higher eukaryotes ((Dowhan, 1997; Voelker, 1997; reviewed by Schuiki and Daum, 2009). To analyze whether TbPSD is proteolytically processed *in vivo*, we expressed a C‐terminally 3xHA‐tagged copy of Tb927.9.10080 in *T. brucei* procyclic and bloodstream forms. Analysis by SDS‐PAGE followed by immunoblotting revealed two bands corresponding to the expected sizes of the tagged proenzyme (i.e. before proteolytic processing) and the α‐subunit after proteolytic cleavage at the predicted recognition site (Fig. 3B). To further study whether the GSS motif of TbPSD is the cleavage site, we expressed a C‐terminally 3xHA‐tagged copy of TbPSD in which Ser318 was replaced by alanine. As shown in Fig. 3, changing the cleavage site from GSS to GAS inhibited processing of the proenzyme, confirming the GSS motif as proteolytic cleavage site of TbPSD.

### TbPSD localizes to the mitochondrion

**Figure 4:**
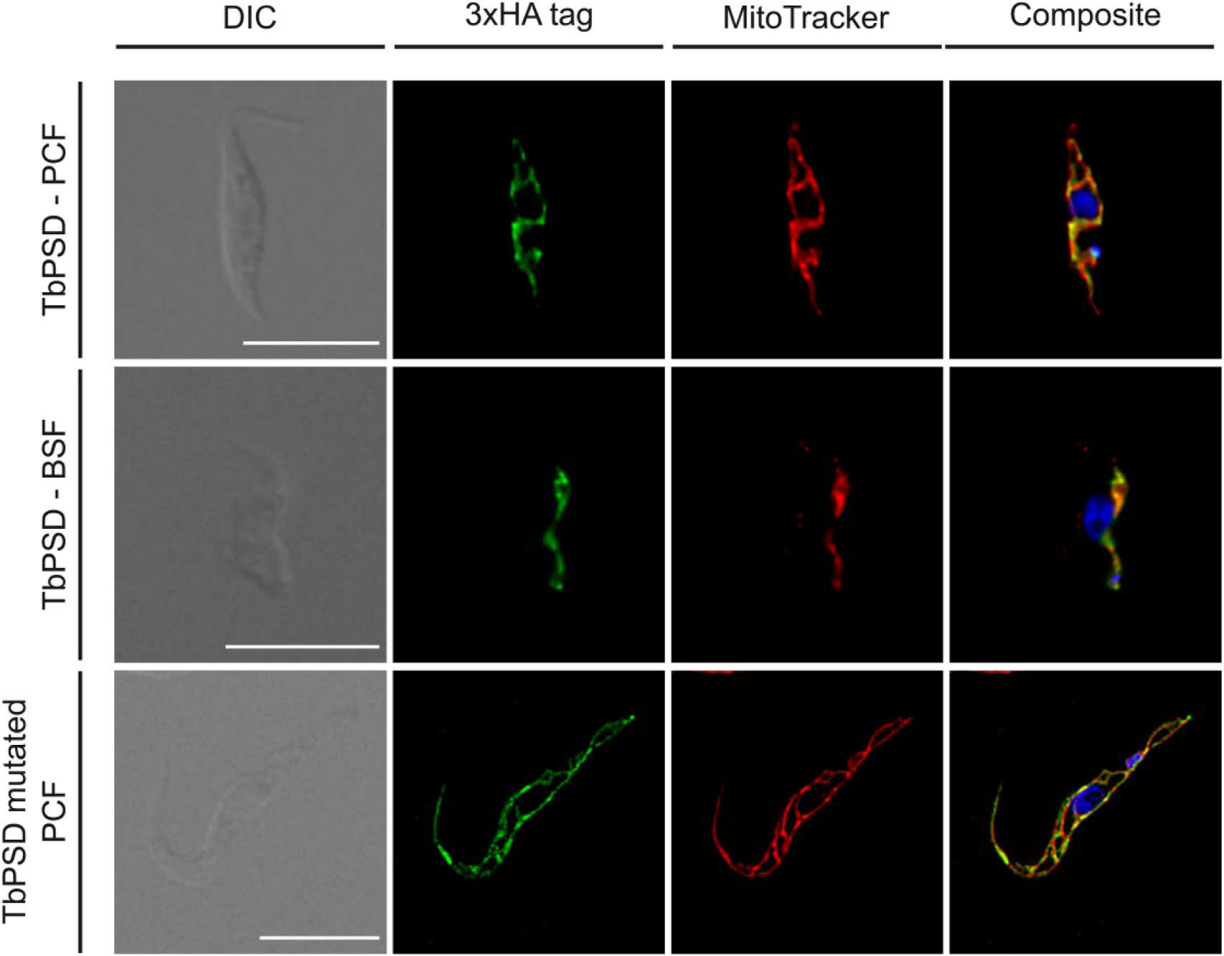
Localization of TbPSD by immunofluorescence microscopy. Hemagglutinin (HA)‐tagged wild‐type (top and middle rows) and mutated forms (bottom row) of TbPSD were expressed in *T. brucei* procyclic (PCF; top and bottom rows) and bloodstream forms (BSF; middle row). Subcellular localization of TbPSD was analyzed by immunofluorescence microscopy using anti‐HA antibody and the mitochondrial dye MitoTracker. DNA (in blue) was stained with 4′,6′‐diamidino‐2‐phenylindole. Scale bars indicate 10 µm.

Analysis by immunofluorescence microscopy showed good co‐localization of TbPSD‐3xHA with MitoTracker, a mitochondrial membrane potential‐dependent red‐fluorescent dye, in both procyclic and bloodstream forms (Fig. 4). Co‐staining with other organelle markers (TbGRASP for Golgi, BiP for ER, Trypanopain for lysosome) showed no co‐localization with TbPSD (results not shown). Mitochondrial localization was also observed for the uncleaved S318A TbPSD‐3xHA mutant (Fig. 4), demonstrating that correct localization of TbPSD is independent of proteolytic processing of the proenzyme into its subunits.

### Down‐regulation of TbPSD inhibits trypanosome growth

**Figure 5:**
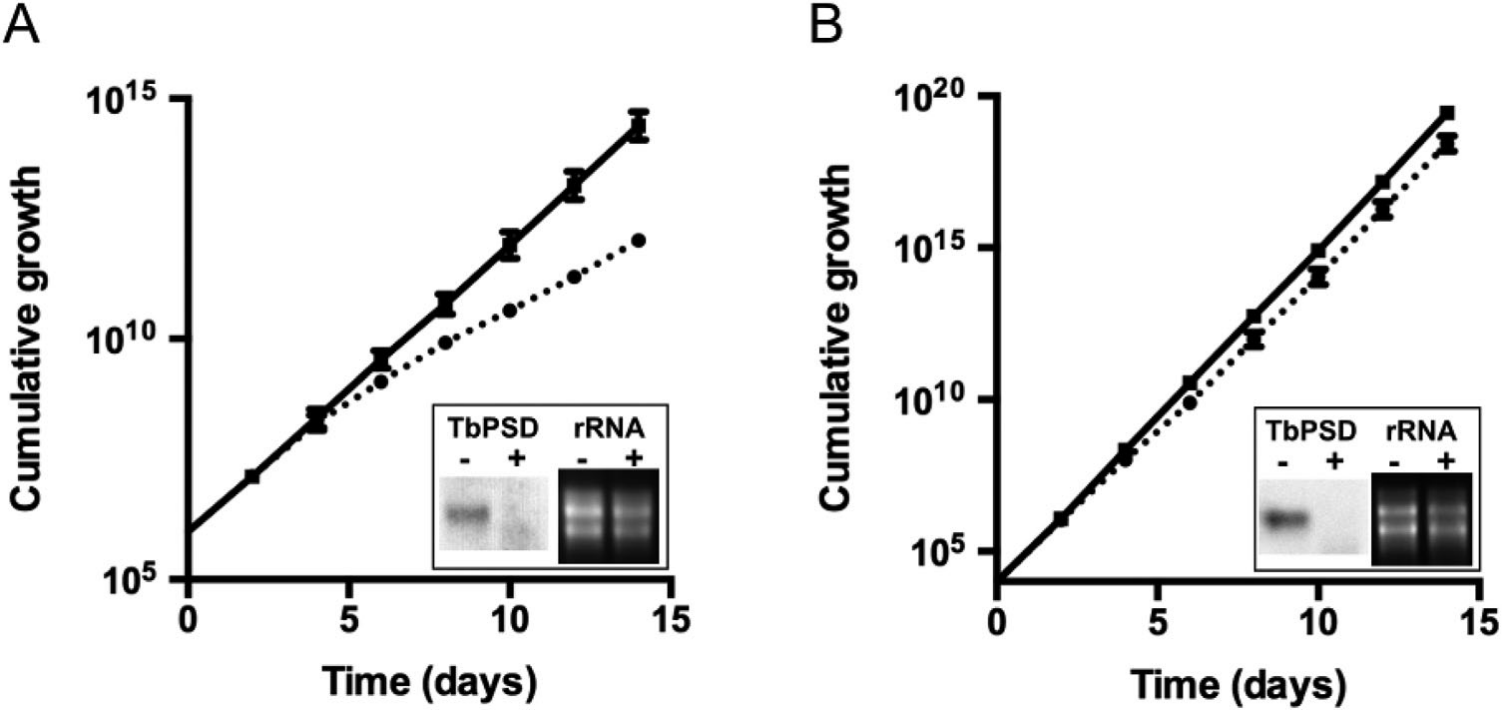
Growth of TbPSD‐depleted parasites. A. Growth of control (solid lines) and TbPSD‐depleted (dotted lines) procyclic forms. The data points represent mean values ± standard deviations from three separate experiments involving two different clones. B. Growth of control (solid lines) and TbPSD‐depleted (dotted lines) bloodstream forms. The data points represent mean values ± standard deviations from three experiments using the same clone. For some data points, the error bars are smaller than the symbols. The insets in A and B show Northern blot analyses of TbPSS2 mRNA levels (left two lanes) in parasites incubated in the absence (−) or presence (+) of tetracycline for 2 days. rRNA levels are shown as loading controls (right two lanes).

Essentiality of TbPSD for *T. brucei* parasites in culture was studied by tetracycline‐inducible RNAi‐mediated gene silencing. Down‐regulation of TbPSD mRNA was analyzed by Northern blotting after 3 days of RNAi induction and showed efficient depletion of TbPSD mRNA in both procyclic and bloodstream form parasites (Fig. 5A and B). After 4 days of RNAi against TbPSD, procyclic forms showed a growth defect compared to control parasites (Fig. 5A), while growth of TbPSD‐depleted bloodstream forms was only slightly affected (Fig. 5B).

### TbPSD depletion affects mitochondrial integrity and ATP production via oxidative phosphorylation

**Figure 6:**
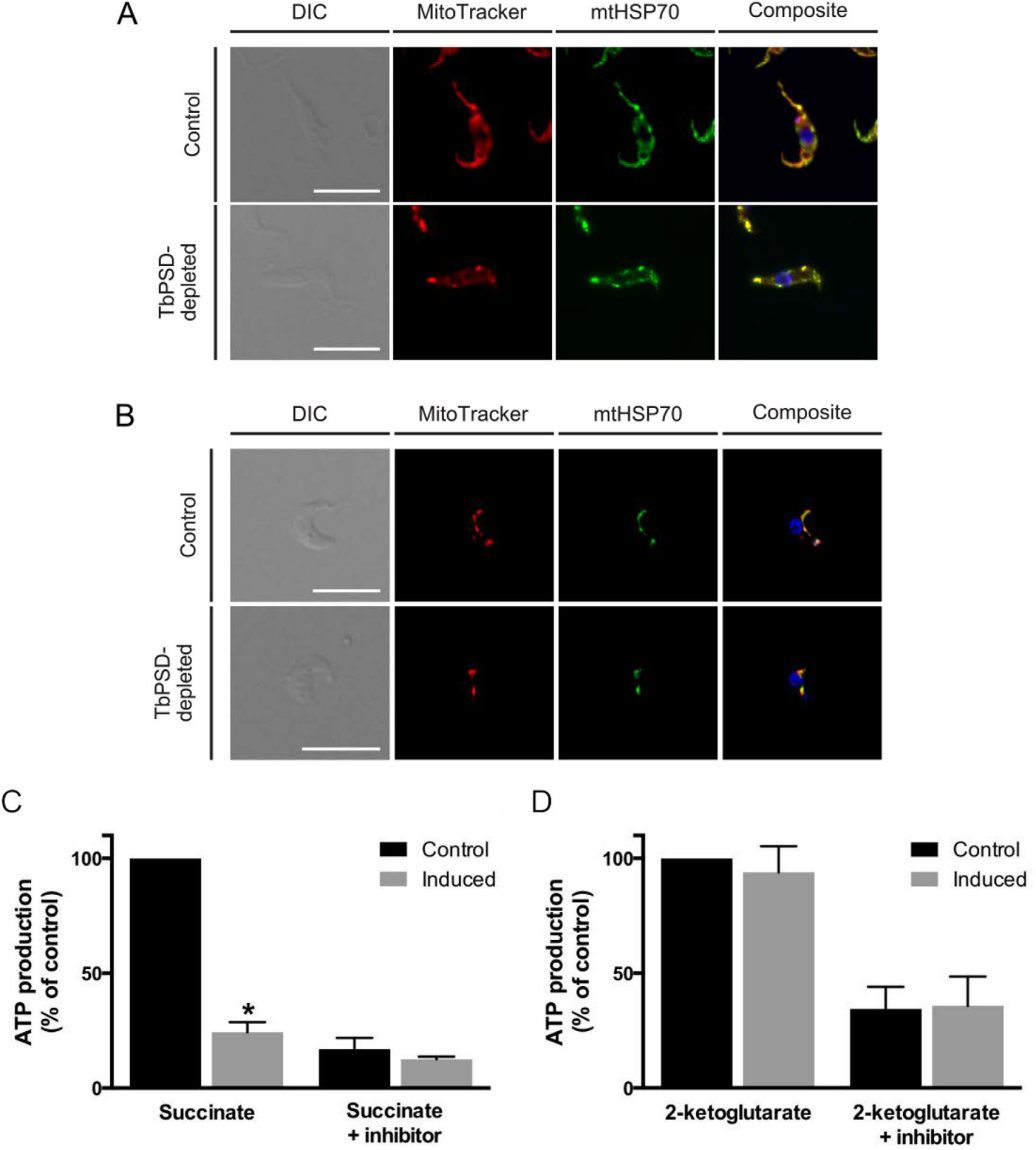
Effect of TbPSD down‐regulation on mitochondria. A, B. Mitochondrial structure and membrane potential were analyzed by immunofluorescence in procyclic (panel A) and bloodstream form (panel B) parasites. Mitochondria from control and TbPSD‐depleted trypanosomes (induced with tetracycline for 6 days) were live‐stained with MitoTracker, then cells were fixed on slides and co‐stained with an antibody against a mitochondrial marker protein (mtHSP70). DNA was stained with 4′,6′‐diamidino‐2‐phenylindole (in blue). Scale bars indicate 10 µm. C, D. ATP production by oxidative phosphorylation (C) or at substrate level (D) was measured in digitonin extracts of control and TbPSD‐depleted trypanosomes (induced with tetracycline for 6 days). Antimycin and atractyloside were added to inhibit complex III (panel C) and ADP/ATP translocase (panel D), respectively. Data are from two independent experiments performed in duplicates. Error bars indicate means ± standard deviations. The asterisk indicates significant difference with control (*P < 0.0001, unpaired student's *t* test).

To study possible effects of TbPSD depletion on mitochondrial integrity, procyclic and bloodstream form trypanosomes were analyzed by fluorescence microscopy. Mitochondria were stained using two different markers: the membrane potential‐dependent dye MitoTracker Red and an antibody against mitochondrial heat shock protein 70 (mtHSP70). While control cells showed the typical branched mitochondrial network in procyclic forms and the tubular mitochondrial structure in bloodstream forms, trypanosomes depleted of TbPSD after 6 days of down‐regulation revealed abnormal staining (Fig. 6A and B). In both life cycle forms, down‐regulation of TbPSD resulted in the appearance of distinct and brightly fluorescent spots, an indication of mitochondrial fragmentation.

Subsequently, we measured mitochondrial ATP production in control and TbPSD‐depleted procyclic forms *in vitro* using digitonin‐solubilized membranes. Succinate was used as substrate to measure ATP formation via oxidative phosphorylation whereas 2‐ketoglutarate was used to determine ATP synthesis via substrate level phosphorylation (Allemann and Schneider, 2000). The results showed a decrease in ATP production via oxidative phosphorylation of more than 75% in TbPSD‐depleted mitochondria isolated from parasites after 6 days of RNAi compared to controls (Fig. 6C). In contrast, ATP production via substrate level phosphorylation was not affected (Fig. 6D).

### PE and PS levels are not affected by down‐regulation of TbPSD

**Figure 7:**
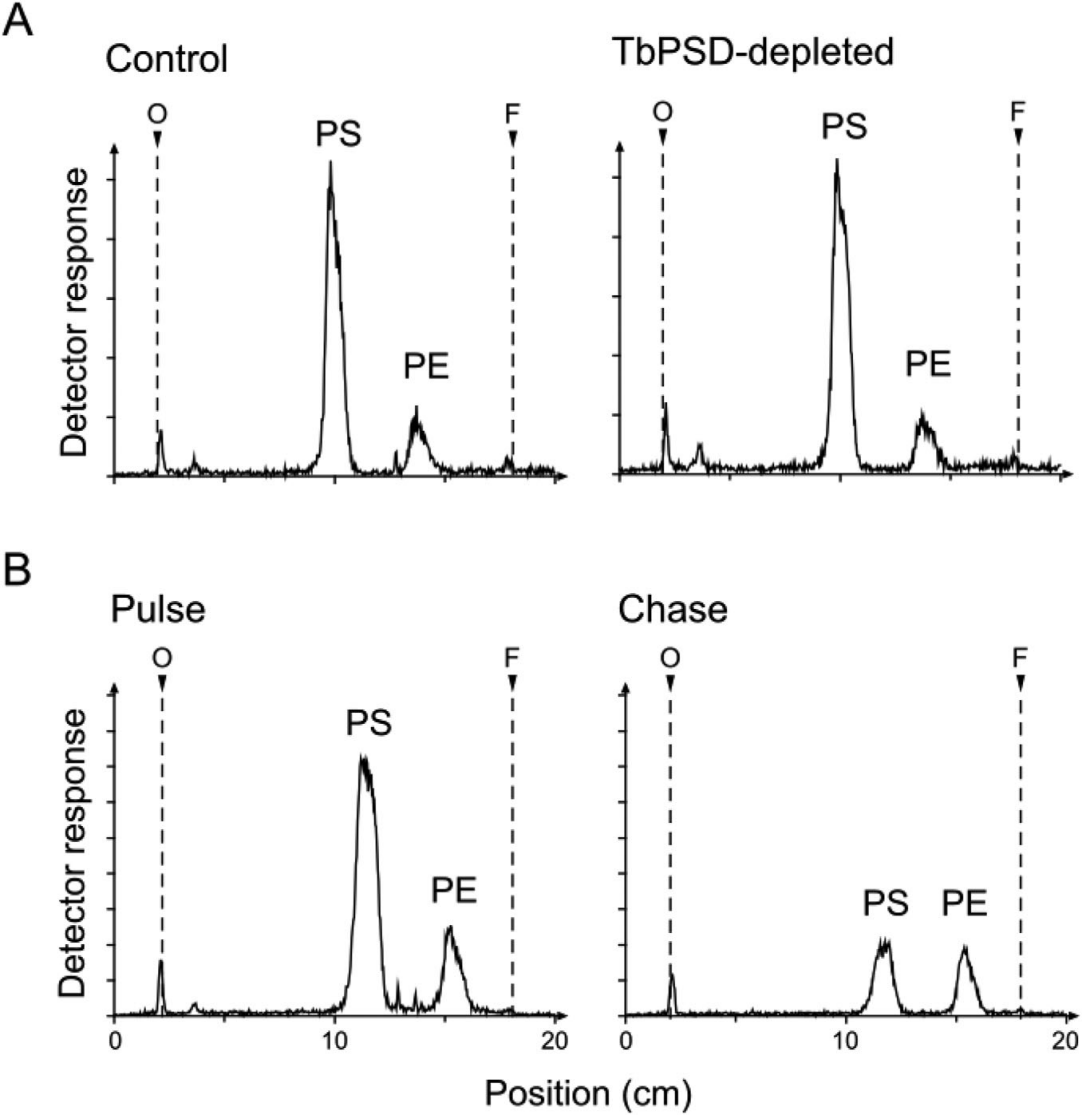
Lipid analysis of TbPSD parasites after [^3^H]serine labeling. Trypanosomes were pretreated with myriocin for 30 min and induced with tetracycline for 6 days to down‐regulate TbPSD. A. Control and TbPSD‐depleted procyclic forms were labeled for 4 h with [^3^H]serine before lipid extraction. B. Control cells were labeled with [^3^H]serine for 4 h (pulse), washed and resuspended in fresh medium without radioactivity for 4 h (chase). Lipids were extracted, separated by one‐dimensional thin layer chromatography and detected using a radioisotope scanner. The scans are representative of at least three independent experiments.

It has previously been suggested that decarboxylation of PS may contribute to PE formation in *T. brucei* procyclic forms (Signorell *et al.*, 2008b). The availability of RNAi parasites against TbPSD now allowed us to experimentally address the importance of this pathway in both procyclic and bloodstream forms. As expected, [^3^H]serine labeling of myriocin‐treated parasites resulted in formation of [^3^H]PS and [^3^H]PE (see also Fig. 2). However, in contrast to our prediction that [^3^H]PE is generated by decarboxylation of [^3^H]PS, we saw no significant difference in [^3^H]PE formation in parasites after down‐regulation of TbPSD (Fig. 7A). The ratios [^3^H]PS/[^3^H]PE and [^3^H]PE/([^3^H]PE+[^3^H]PS) in parasites after RNAi against TbPSD were unchanged compared to control uninduced cells (5.5 ± 1.3 vs. 6.0 ± 1.0 and 15.6 ± 2.8 vs. 14.5 ± 2.0 respectively; mean values ± standard deviations from three independent experiments). In addition, we saw no major differences in total phospholipid composition between control and TbPSD‐depleted parasites after analysis of lipid extracts by two‐dimensional TLC and phosphorus quantification (Supporting Information Fig. S4). Based on our findings that RNAi against TbPSD induces changes in mitochondrial structure, affects ATP production (Fig. 4) and reduces procyclic parasite growth (Fig. 5), we think it is unlikely that [^3^H]PE formation in TbPSD‐depleted cells is due to residual amounts of TbPSD activity in these parasites. Alternatively, we hypothesize that [^3^H]PE may be formed by a reaction sequence that is independent from TbPSD, i.e. via decarboxylation of [^3^H]serine to [^3^H]ethanolamine followed by synthesis of [^3^H]PE by the Kennedy pathway. Such an interpretation is in line with the above‐mentioned observation that digitonin‐solubilized membranes readily form [^3^H]PS *in vitro* during incubation with [^3^H]serine, but are unable to convert [^3^H]PS to [^3^H]PE by mitochondrial TbPSD (Fig. 2C and D).

Interestingly, we observed that the amounts of [^3^H]PS formed during labeling with [^3^H]serine decreased during a subsequent 4 h chase in the absence of [^3^H]serine (Fig. 7B). In contrast, labeling of [^3^H]PE remained unchanged during the chase (Fig. 7B), resulting in a change in the [^3^H]PS/[^3^H]PE ratio from 2.61 at the end of the pulse to 1.15 after the chase period (mean values from five independent experiments). As a similar change in the [^3^H]PS/[^3^H]PE ratio was also observed in TbPSD‐depleted parasites, it is unlikely caused by TbPSD‐mediated conversion of [^3^H]PS to [^3^H]PE. Alternatively, the loss of [^3^H]PS during the chase may be due to TbPSS2‐mediated conversion of [^3^H]PS to PE, with concomitant loss of the label.

Together, [^3^H]serine‐labeling experiments of TbPSD RNAi parasites and digitonin‐solubilized membranes demonstrated that in *T. brucei* [^3^H]PE is not formed from [^3^H]serine via [^3^H]PS and subsequent decarboxylation to [^3^H]PE, but instead involves a previously unrecognized reaction sequence. Since plant cells (Rontein *et al*., 2001) and *Plasmodium* parasites (Elabbadi *et al*., 1997) are capable of decarboxylating serine to ethanolamine via serine decarboxylase, we investigated if a putative *T. brucei* serine decarboxylase activity may convert [^3^H]serine to [^3^H]ethanolamine, which would then enter the Kennedy pathway to form [^3^H]PE. However, using previously published protocols (Elabbadi *et al*., 1997), we were unable to detect such an activity in *T. brucei* procyclic forms. In addition, we found no candidate gene in the *T. brucei* genome with homology to plant serine decarboxylase. It should be noted that the gene responsible for the observed serine decarboxylase activity in *Plasmodium* has not been reported.

### TbPSD is functional in *E. coli*

**Figure 8:**
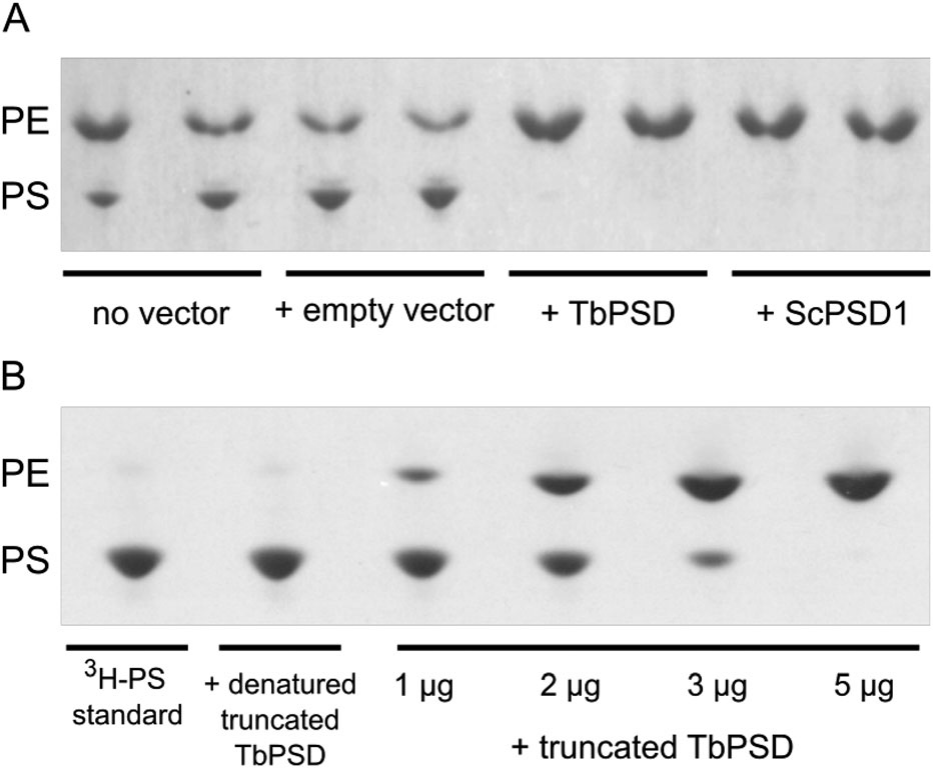
Recombinant PS decarboxylase activity in *E. coli*. A. Membranes from *E. coli* containing either no vector, the empty vector or the vector containing TbPSD or *S. cerevisiae* PSD1 (ScPSD1) for complementation were incubated with [^3^H]PS for 10 min. After extraction, lipids were analysed by high performance thin layer chromatography and radiolabeled PS and PE were detected by fluorography. Samples from two parallel experiments were applied. B. Soluble (truncated) TbPSD was expressed and purified from *E. coli* and assayed with [^3^H]PS for 60 min in the same manner as the full length enzyme. Lipids were extracted and analysed as in panel A. The migration of PE and PS standards applied on the same plate is indicated in the left margins.

To study whether Tb927.9.10080 encodes a functional PS decarboxylase, the full‐length form was recombinantly expressed in *E. coli*. [^3^H]PS was then added to a membrane preparation and incubated for 10 min. Subsequently, lipids were extracted and separated by HPTLC and radiolabeled lipids visualized by fluorography. As seen in Fig. 8A, in the presence of either no vector or empty vector, there is some conversion of [^3^H]PS to [^3^H]PE, due to the endogenous *E. coli* PSD activity. In the presence of expressed TbPSD and *S. cerevisiae* PSD within this membrane background all of the [^3^H]PS is converted to [^3^H]PE, suggesting additional PSD activity that can be attributed to the expressed PSD homologs. To assay recombinant TbPSD in the absence of endogenous *E. coli* PSD, we expressed and purified a soluble form of TbPSD lacking the N‐terminal membrane domain in *E. coli*. As shown in Fig. 8B, soluble truncated TbPSD was active and readily converted [^3^H]PS to [^3^H]PE in a concentration‐dependent way. Together, these results demonstrate that TbPSD is active when expressed in *E. coli*.

### TbPSD is not functional in *S. cerevisiae*

To test whether TbPSD enzyme is expressed and functional in yeast, multiple constructs of TbPSDs controlled by the ADH1 promoter were introduced into the *S. cerevisiae psd1Δpsd2Δdpl1Δ* strain. The constructs include (1) a full length TbPSD, (2) N‐terminal deletion mutants of TbPSD (Δ48 and Δ83) and (3) a chimera TbPSD where the N‐terminal 139 amino acids of the yeast PSD1 mitochondrial targeting sequence are fused to Δ83TbPSD. The yeast mutant strains are devoid of functional PSD enzymes and require ethanolamine for growth. Expression of the various constructs of TbPSD in the mutant strain failed to suppress the growth defect in the absence of exogenous ethanolamine (Supporting Information Fig. S5). The results indicate that none of the TbPSD forms were functional when expressed in yeast.

In addition, we expressed V5‐tagged full length TbPSD and Sc‐TbPSD chimera using pYES2.1 vector under the strong GAL1 promoter in *psd1Δpsd2Δdpl1Δ* strain. Similar to the findings above, the V5‐tagged constructs also failed to suppress the growth defect (Supporting Information Fig. S6). Immunoblot analysis of cell extracts containing TbPSD‐V5 fusion and Sc‐TbPSD‐V5 chimera using anti‐V5 antibody show that TbPSD pro‐enzymes can be detected, however the signals were weak compared to a yeast PSD1‐V5 control (Supporting Information Fig. S6). Interestingly, the processed alpha subunits were barely detected, indicating poor processing of the pro‐enzyme into its active form. Together, these results indicate that TbPSD constructs were not functional in yeast because they were poorly expressed and not effectively processed.

## Discussion

Although the multiple pathways for PE biosynthesis are conserved among organisms, their contributions vary significantly. Several eukaryotes and most prokaryotes use decarboxylation of PS as main route for PE synthesis, while mammalian cells use both PS decarboxylation and the CDP‐ethanolamine branch of the Kennedy pathway for PE formation. *T. brucei* parasites are unique in that the Kennedy pathway represents the only pathway for *de novo* production of PE (reviewed by Farine and Bütikofer, 2013). Its disruption, by depletion of any of the four enzymes catalyzing the individual reactions, leads to inhibition of growth and parasite death (Gibellini *et al*., 2008; Signorell *et al.*, 2008b; Gibellini *et al*., 2009; Signorell *et al*., 2009). The presence of PS decarboxylase and PS synthase activities in *T. brucei* has been suggested in several reports (Menon *et al*., 1993; Rifkin *et al*., 1995; Signorell *et al.*, 2008b), but TbPSD activity was later shown to be absent in bloodstream trypanosomes (Gibellini *et al*., 2009; Richmond *et al*., 2010). However, the enzymes were not characterized biochemically and the contributions of these pathways to PE formation and their importance for parasite viability were not investigated. We now report the identification and characterization of TbPSS2 and TbPSD. By using tetracycline‐inducible RNAi against the individual enzymes, we demonstrate that their expression in *T. brucei* is essential for normal parasite growth in culture.

Our results using *in vivo* and *in vitro* [^3^H]serine labeling demonstrate that depletion of TbPSS2 in procyclic form trypanosomes completely blocks *de novo* formation of PS and reduces cellular PS levels, identifying TbPSS2 as the sole route for PS synthesis in *T. brucei*. PSS enzymes belong to two categories: in bacteria and yeast, PSS catalyzes PS formation using serine and CDP‐DAG as substrates, whereas in mammals and plants PS is formed via head group exchange (reviewed by Schuiki *et al*., 2010; Vance and Tasseva, 2013). Thus, our identification of a base exchange enzyme in the ancient eukaryote *T. brucei* is remarkable and is in accordance with recent results from *T. brucei* CDP‐DAG knockout trypanosomes showing that PS production is largely unaffected after knocking down CDP‐DAG synthesis (Lilley *et al*., 2014). Comparisons of the primary sequence of TbPSS2 with its predicted homologs in other kinetoplastids, including *T. cruzi* and *Leishmania* spp., indicate that PS synthesis in these parasites is also catalyzed by a head group exchange enzyme (Ramakrishnan *et al*., 2013).

Mammalian PSS1/2 enzymes have been localized to mitochondria‐associated membranes (MAMs) (Stone and Vance, 2000). Enzyme activities were found in the ER and enriched in MAMs, while immunofluorescence studies using c‐myc‐tagged PSS1/2 showed co‐localization with an ER marker (Stone and Vance, 2000). Consistent with the results in mammalian cells, using immunofluorescence microscopy we localized a C‐terminally 3xc‐myc‐tagged copy of TbPSS2, which was shown to be catalytically active, in the ER of *T. brucei* procyclic forms. At present, it is not known whether MAMs are also present in *T. brucei* parasites.

Incubation of *T. brucei* parasites with [^3^H]serine has been shown to not only label sphingolipids and PS, but also PE (Menon *et al*., 1993; Signorell *et al.*, 2008b). Conversion of PS to PE has been observed in many organisms before and has been attributed to PSD enzymes (Kanfer and Kennedy, 1964; Butler and Morell, 1983; Vance and Vance, 1986; Trotter and Voelker, 1995; Elabbadi *et al*., 1997; Choi *et al*., 2012). Our results demonstrate that *T. brucei* parasites express a mitochondrial type I PSD enzyme, TbPSD, which undergoes similar proteolytic processing as its mammalian (Kuge *et al*., 1996) and yeast (Horvath *et al*., 2012) homologs. RNAi‐mediated down‐regulation of TbPSD affected mitochondrial morphology in both procyclic and bloodstream forms, reduced ATP production via oxidative phosphorylation in procyclic trypanosomes and reduced parasite growth in culture. When expressed in *E. coli*, but not in *S. cerevisiae*, TbPSD was active. However, as reported before for *T. brucei* bloodstream forms (Gibellini *et al*., 2009), we were unable to demonstrate *in vitro* or *in vivo* PS decarboxylation activity in procyclic forms. Although labeling of parasites with [^3^H]serine always lead to production of radiolabeled PE, suggesting the presence of a PS decarboxylation activity converting [^3^H]PS to [^3^H]PE, the reaction was not decreased in parasites after depletion of TbPSD. In addition, a mitochondrial extract competent for synthesizing [^3^H]PS was unable to generate [^3^H]PE. Together, these results indicate that using [^3^H]serine as substrate, [^3^H]PE in *T. brucei* is unlikely formed via PS followed by its decarboxylation to PE, but by a different mechanism involving soluble factors that are lost upon extraction of parasites with digitonin. The presence of a serine decarboxylase activity in *T. brucei* could explain our findings, however, we were unable to detect such an activity using published procedures (Elabbadi *et al*., 1997). In addition, mining the *T. brucei* predicted proteome with plant‐type serine decarboxylases revealed no reasonable sequences. Since TbPSD is essential in *T. brucei*, we hypothesize that it may be involved in limited and locally restricted synthesis of PE in the mitochondrion, perhaps by producing a subset of PE molecular species important for mitochondrial structure and function, whereas bulk mitochondrial PE may be generated via the Kennedy pathway. Alternatively, we can't exclude the possibility that TbPSD acts on a different substrate from PS, such as phosphatidylthreonine as recently identified in *T. gondii* (Arroyo‐Olarte *et al*., 2015).

## Experimental procedures

Unless otherwise stated, all reagents were of analytical grade and purchased from Sigma‐Aldrich (Buchs, Switzerland) or Merck (Darmstadt, Germany). Restriction enzymes were obtained from Fermentas (St. Leon‐Rot, Germany) and antibiotics from Sigma‐Aldrich, Invivogen (Nunningen, Switzerland) or Invitrogen (Basel, Switzerland). [^3^H]Serine (1 mCi ml^−1^, 20 Ci mmol^−1^) was purchased from American Radiolabeled Chemicals (St. Louis, USA) and [γ‐^32^P]‐dCTP (3000 Ci mmol^−1^) from PerkinElmer (Waltham, USA). Phospholipid standards were purchased from Sigma‐Aldrich or Avanti Polar Lipids Inc. (Alabaster, AL).

### Trypanosomes and culture conditions

*T. brucei* procyclic forms expressing a T7 polymerase and a tetracycline repressor (obtained from Paul Englund, Baltimore) (Wirtz *et al*., 1999) were cultured at 27°C in SDM‐79 (Brun and Schönenberger, 1979) containing 10% heat‐inactivated fetal bovine serum (FBS, Invitrogen), 25 µg ml^−1^ hygromycin and 15 µg ml^−1^ G418. Bloodstream New York single‐marker trypanosomes were maintained at 37°C, 5% CO_2_ in HMI‐9 containing 10% FBS and 1 μg ml^−1^ G418.

### Generation of 3xHA‐tagged TbPSD and 3xc‐myc‐tagged TbPSS2

To generate C‐terminally 3xHA‐tagged TbPSD (Tb927.9.10080), the corresponding open reading frame (ORF) was amplified by polymerase chain reaction (PCR) using primers PSD_HA_Fw GCCCAAGCTTATGGCTTCGCTCACACGACAACTGC and PSD_HA_Rv CGGCTCTAGACTTGCCACTCTCCGACAACCCGTAT (restriction sites underlined). The PCR product was ligated into the *Hind*III‐ and *Xho*I‐ digested plasmid pAG3020‐3 (Gonzalez‐Salgado *et al*., 2012) resulting in plasmid pLF1610HA. To produce N‐terminally 3xc‐myc‐tagged TbPSS2, the corresponding ORF (Tb927.7.3760) was amplified using primers PSS2pJM2_Fw GCGCTCGAGGCCGGTAAGCTCAACGGTGCTACCG and PSS2pJM2_Rv CGGGATCCTTATCGCCAAAAGATTATGTAGCGCTCGTAT and ligated into plasmid pJM2 (Desy *et al*., 2012), resulting in plasmid pLF3760cmyc. Before transfection into trypanosomes, plasmids were linearized using NotI enzyme to allow proper integration into the genome of the parasite.

### RNAi‐mediated gene silencing

Putative TbPSD mRNA was down‐regulated in procyclic and bloodstream trypanosomes by RNAi‐mediated gene silencing using stem‐loop constructs containing a puromycin resistance. A 374 bp fragment containing the last 361 bp of the TbPSD ORF and the first 13 bp of the TbPSD 3′UTR was amplified by PCR using primers PSD_RNAi_Fw GCGCCCAAGCTTGGATCCCTCTGTCCTTCCATTAAACG and PSD_RNAi_Rv CTAGGCTCTAGACTCGAGTTGTGCATACTGCCTACTTG (restriction sites underlined) and cloned into the tetracycline‐inducible vector pMS14 (Serricchio and Bütikofer, 2013) resulting in plasmid pLF1610RNAi. For PSS2 RNAi in procyclic forms, a 550 bp fragment of the TbPSS2 ORF was amplified using primers fwd3760 GTGAAGCTTGGATCCACGATTATTGTGAGGGATTG and rev3760 GTGAGATCTGAGCTCACAAAAGGTTCACCACACTC and cloned into the tetracycline‐inducible vector pALC14 resulting in plasmid pJJ3760RNAi. Selection of the gene sequence for RNAi was done with RNAit, a prediction algorithm designed to prevent potential cross‐talk and hence off‐target effects (Redmond *et al*., 2003). Before transfection into trypanosomes, plasmids were linearized using *Not*I enzyme.

### Stable transfection of trypanosomes

Trypanosomes were harvested at mid‐log phase, washed once in phosphate‐buffered saline (PBS; 137 mM sodium chloride, 2.7 mM potassium chloride, 10 mM disodium phosphate, 2 mM monopotassium phosphate, pH 7.4) and suspended in 100 µl TbBSF buffer (90 mM sodium phosphate, 5 mM potassium chloride, 0.15 mM calcium chloride, 50 mM HEPES, pH 7.3) (Schumann Burkard *et al*., 2011) previously mixed with 10 µg of linearized plasmid (pLF1610HA, pLF1610RNAi, pJJ3760RNAi and pLF3760cmyc). Electroporation was performed in 100 µl Nucleocuvette using Lonza 4D Nucleofector System (pulse code FI‐115, “Primary Cell P3” solution). Recombinant clones were obtained by limited dilutions and selected with 1.75 µg ml^−1^ phleomycin for pLF1610RNAi or 2 µg ml^−1^ puromycin for the other vectors. Proper integration of the constructs was confirmed by PCR using primers binding upstream of the recombination sites and at the end of the inserted genes. Expression of tagged proteins or generation of double‐stranded RNA was induced by addition of 1 µg ml^−1^ tetracycline to the culture medium.

### Northern blot analysis

Total RNA was extracted from 4 x 10^7^ procyclic or bloodstream form parasites using the Total SV RNA extraction Kit (Promega). 20 μg of RNA were loaded and run on a 1% agarose gel. The gel was stained with ethidium bromide and rRNA amounts were assessed as loading control. After transfer onto a Hybond‐N^+^ nylon membrane (Amersham Pharmacia Biotech) using 10x SSC buffer (150 mM trisodium citrate, pH 7.0, containing 1.5 M sodium chloride), RNA was cross‐linked by UV irradiation. The membrane was probed with a [^32^P]‐labeled 374 bp probe of the TbPSD ORF/3'UTR or a [^32^P]‐labeled 550 bp probe of the TbPSS2 ORF, generated using the Prime‐a‐Gene labeling system (Promega). Detection was done by autoradiography using BioMax MS films (GE Healthcare, Buckinghamshire, UK) in combination with intensifying screens.

### (Immuno‐) Fluorescence microscopy

For MitoTracker staining, 1 × 10^7^ procyclic or 2 × 10^6^ bloodstream form trypanosomes were incubated for 30 min with 250 mM MitoTracker Red CM‐H_2_XRos (Invitrogen) in culture medium. After washing, parasites were spread and allowed to adhere to a microscopy slide, fixed with 4% paraformaldehyde, air dried and mounted with Vectashield containing DAPI (4′,6′‐diamidino‐2‐phenylindole, Vector Laboratories). Alternatively, MitoTracker‐stained and paraformaldehyde‐fixed cells were used for co‐localization studies using the following immunofluorescence protocol.

For immunofluorescence microscopy, trypanosomes were collected and washed before being allowed to adhere onto a microscopy slide (Thermo Scientific) for 10 min. Cells were fixed with 4% paraformaldehyde, washed 3× with cold PBS and permeabilized with 0.2% Triton X‐100 in PBS. Blocking was performed with 2% bovine serum albumin in PBS for 30 min followed by 45 min incubation of first antibody diluted in blocking solution. The following antibodies were used: mouse monoclonal α‐HA.11 16B12 (Covance, 1:250 dilution), mouse α‐c‐myc (Santa Cruz Biotech, 1:200 dilution) or rabbit α‐c‐myc (Bethyl Laboratories, 1:200 dilution), mouse α‐mtHSP70 (kindly provided by Paul Englund, Baltimore, 1:1000 dilution) and rabbit α‐BiP (kindly provided by Jay Bangs, Buffalo, 1:2000 dilution). After washing, secondary antibodies goat anti‐mouse and anti‐rabbit AlexaFluor 594 and 488 (Invitrogen, 1:800 dilution in blocking solution) were added for 45 min in either combinations. After washing and air‐drying, cells were mounted with Vectashield containing DAPI. Fluorescence microscopy was performed with a Leica DMI6000 B inverted microscope using a 60× oil objective. Pictures were acquired, processed and 3D‐deconvolved with the Leica LAS AF Version 2.1.0 software (Leica Microsystems CMS GmbH).

### SDS‐PAGE and immunoblotting

Extracted proteins were separated on 12% polyacrylamide gels under reducing conditions (Laemmli, 1970). Proteins were transferred on Immobilon‐P polyvinylidene fluoride membranes (Millipore, USA) by semi‐dry blotting. After blocking in TBS buffer (10 mM Tris‐HCl pH 7.5, 144 mM NaCl) containing 5% (wt/vol) milk powder, membranes were exposed to α‐HA mouse monoclonal primary antibody (1:3000). Horseradish peroxidase‐conjugated secondary antibody anti‐mouse (Dako, Agilent Technologies) was used at a concentration of 1:5000 and detected using an enhanced chemiluminescence detection kit (Pierce).

### [^3^H]Serine labeling and lipid analysis

Procyclic form trypanosomes (5–10 × 10^7^) were pre‐treated with 2.5 µM myriocin for 30 min and labeled with 30 μCi [^3^H]serine for 4 h. For pulse/chase experiments, parasites labeled for 4 h were collected, washed once and resuspended in fresh medium without radioactivity. The chase was conducted for 2 to 6 h. Lipids were extracted according to the work by Bligh and Dyer (1959) and separated by one‐dimensional thin layer chromatography (TLC) (Silica gel 60 plate, Merck) using solvent system I (chloroform: methanol: acetic acid: water (25:15:4:2, by vol.)). Products from the aqueous phase were resolved using solvent system II (0.6% NaCl in water: methanol: 25% ammonium hydroxide (10:10:1, by vol.)). Radioactive standards were run on each plate alongside the samples to be analyzed. Radioactivity was detected by scanning the dried plate with a radioisotope detector (Berthold Technologies) and quantified using the Rita Star software provided by the manufacturer. For two‐dimensional TLC followed by lipid phosphorus quantification or fluorography, 4 × 10^8^ procyclic trypanosomes were labeled overnight with 60 μCi [^3^H]serine, lipids were extracted and separated with solvent system III (chloroform:methanol:25% ammonium hydroxide:water (90:74:12:8, by vol.)) for the first dimension and solvent system IV (chloroform:methanol:acetone:acetic acid:water (40:15:15:12:8, by vol.)) for the second dimension. Radioactive plates were dried, sprayed with EN^3^HANCE Spray (PerkinElmer) and exposed to BioMax MS films (GE Healthcare, Buckinghamshire, UK) for 3 days at −75°C. For quantification of lipid using phosphorus determination, individual spots were scraped from TLC plates and quantified as described before (Signorell *et al.*, 2008a).

### Preparation and labeling of membranes‐containing digitonin extracts

Digitonin extracts were prepared as described elsewhere (Schneider *et al*., 2007). Procyclic form trypanosomes (1 × 10^8^ cells) were harvested, washed once in SBG buffer (150 mM Tris‐HCl pH 7.9, 20 mM glucose, 20 mM NaH_2_PO_4_) and suspended in SoTE buffer (20 mM Tris‐HCl pH 7.5, with 600 mM sorbitol and 2 mM EDTA) containing a final concentration of 0.025% (wt/vol) digitonin. After 5 min of incubation on ice, mitochondria were isolated by centrifugation at 6200*g*. After removal of the supernatant, membranes from digitonin extract were resuspended in 500 μl SoTE and labeled with 15 μCi [^3^H]serine for 4 h. Lipids were extracted and analyzed as described above.

### ATP production assay

ATP production in digitonin extracts was measured as previously described (Allemann and Schneider, 2000). About 5 mM of succinate or 2‐ketoglutarate and 67 μM of ADP were added to membranes isolated by digitonin extraction from 9.5 × 10^7^ procyclic trypanosomes and incubated for 30 min at room temperature. Antimycin (inhibitor of complex III) and atractyloside (inhibitor of ADP/ATP translocase) were pre‐incubated with digitonin extracts for 10 min at a concentration of 2.7 and 43 μM, respectively. ATP concentrations were determined using ATP Bioluminescence Assay Kit CLS II (Roche, Basel, Switzerland).

### Recombinant expression and enzyme activity of TbPSS2 in *E. coli*

The TbPSS2 ORF was obtained by PCR from *T. brucei* gDNA, using 5′‐CGCGGATCCGATGGCCGGTAAGCTCAAC‐3′ and 5′‐CCGCTCGAGTCGCCAAAAGATTATGTA‐3′ primers. The PCR product was sub‐cloned into pET32b expression vector, expression was induced in *E. coli* (BL‐21 strain) at 15°C overnight with 0.5 mM isopropyl β‐d−1‐thiogalactopyranoside. *E. coli* membranes were isolated and PSS activity assays conducted, using an empty vector expressing *E. coli* as a control. PSS activity was assayed as follows. 100 mM potassium phosphate (pH 7, 5), 20 mM MgCl_2_, 1% (wt/vol) Triton X‐100, 50 µg membrane protein and 0.05 µCi [^3^H]serine (ARC, specific activity 30.5 Ci mmol^−1^) with or without 300 μM substrate (PE, PC, PS, PI, DAG, CDP‐DAG; all dipalmitoyl) in a final volume of 200 μl. After sonication reactions were incubated at 30°C for 1 h, and terminated by the addition of 750 μl methanol: chloroform (2:1, by vol). Lipids were extracted in the organic lower layer after making the extract biphasic by the addition of 250 μl of water and chloroform_,_ allowing the incorporation of the radiolabel to be quantitated by liquid scintillation counting.

### TbPSD activity assay in *E. coli*

The TbPSD and *ScPSD1* ORF were obtained by PCR from gDNA and cDNA respectively, using the TbPSD primers 5′‐CGCGGATCCGATGGCTTCGCTCACACGACAACT‐3′ (forward for full length TbPSD) or 5′‐CGCGGATCCGCGGTACCAACTTGCCGCA‐3′ (forward for truncated TbPSD) and 5′‐CCGCTCGAGCTTGCCACTCTCCGACAA‐3′ (reverse), while for the *ScPSD1* ORF 5′‐CGCGGATCCGATGTCAATTATGCCAGTTAAGAACG‐3′ and 5′‐CCGCTCGAGTTTTAAATCATTCTTTCCAATTA‐3′ primers were used. The PCR products were digested and ligated into pET 32b using *BamH*I and *Xho*I. TbPSD expression was induced with IPTG (0.05 mM) in BL21 (DE3) at 15°C overnight and confirmed by immunoblot analysis. The full length PSD assay utilized a membrane preparation (100 µg membrane protein) from either BL‐21 cells only or with empty pET 32b or pET 32b‐TbPSD or pET 32b‐ScPSD. Assay includes 100 mM Hepes (pH 7, 4), 20 mM MgCl_2_, 0.3% *n*‐octyl‐glucopyranoside and 0.1 µCi [^3^H]PS (specific activity 60 Ci mmol^−1^). Reactions were performed at 30°C for 10 min. After termination, lipids were extracted and separated on high performance thin layer chromatography silica plates in chloroform:methanol:water (65:25:4, by vol), radiolabeled PS and PE were detected by fluorography.

The truncated soluble form of TbPSD was expressed in the same manner as the full length enzyme but then purified using Ni^2+^ affinity purification. Various amounts of protein were assayed in the same way as above except reactions were performed at 30°C for 60 min.

### Construction of TbPSD vectors and complementation of the yeast psd1Δpsd2Δdpl1Δ strain

Constitutive expression vectors harboring a full length TbPSD and truncated TbPSDs (lacking the first 48 or 83 amino acids) were made using the *pBEVY‐U* vector (Miller, 1998). Briefly, specific primers for the individual constructs were generated and used to amplify DNA from pALC14 vector harboring TbPSD using PCR with Phusion High‐Fidelity DNA polymerase (New England Biolabs Inc.). The TbPSD cDNA constructs also contained 5′ and 3′ flanking sequences that are homologous to those of the vector. The constructs were introduced into a linearized *pBEVY‐U* vector (by digestion with *Kpn*I and *EcoR*I) through ligation reactions by In‐Fusion HD Cloning Kits (Clontech Laboratories Inc.) to yield *pBEVY‐U‐TbPSD, pBEVY‐U‐* ***Δ*** *48TbPSD*, and *pBEVY‐U‐* ***Δ*** *83TbPSD*. To construct *pBEVY‐U‐Chimera‐Δ83TbPSD* vector harboring a chimera of *ScPSD1‐TbPSD* in which 139 amino acids of the yeast N‐terminus *PSD1* mitochondrial targeting sequence were fused to ***Δ*** *83TbPSD*, the DNA fragment of the N‐terminus *ScPSD1* sequence was amplified by PCR and introduced into the linearized *pBEVY‐U‐* ***Δ*** *83TbPSD* (by *Kpn*I digestion) through the ligation reaction by the In‐Fusion HD Cloning kits. All TbPSD sequences in the newly made constructs were confirmed by DNA sequencing. For complementation, plasmids were transformed into an ethanolamine auxotrophic strain, HKY44 (*MATalpha psd1‐Δ1::TRP1 psd2‐Δ1::HIS3 dpl1Δ::KanMX trp1 ura3 his3 lys2 leu2*) (Storey *et al*., 2001) by using Yeastmaker yeast transformation kit (Clontech Laboratories Inc.). The transformants were selectively grown on uracile dropout synthetic glucose (SC‐U) plates supplemented with 2 mM ethanolamine. The transformants were replica plated into the SC‐U plates supplemented without ethanolamine and the plates with ethanolamine.

A galactose inducible *pYES2.1‐V5* vector was used to express a full length TbPSD, a V5‐tagged full length TbPSD and a V5‐tagged chimera of *ScPSD1‐TbPSD* in yeast *psd1Δpsd2Δdpl1Δ* strain. Specific primers were generated so that proteins could be expressed as untagged or C‐terminal V5 fusion forms. The PCR constructs were amplified from vector templates *pBEVY‐U‐TbPSD* and *pBEVY‐U‐Chimera‐Δ83TbPSD*. The constructs were ligated into a linearized pYES2.1‐V5 TOPO vector (Invitrogen) and transformed into ethanolamine auxotrophic PTY44 strain. The transformants were selectively grown on uracile dropout synthetic galactose (SG‐U) plates supplemented with 2 mM ethanolamine. The transformants were replica plated into the SG‐U plates supplemented without ethanolamine and the plates with ethanolamine.

### TbPSD activity assay in trypanosomes

About 100 million trypanosomes were suspended in 500 µl of extraction buffer solution (50 mM Tris‐HCl, pH 8, 10 mM 2‐mercaptoethanol, 1 mM EDTA, 0.25 M sucrose, 0.5 mM PMSF, Sigma Protease inhibitor cocktail) and the cell free extracts were obtained by sonication (30 sec burst at 12% amplitude, 3 times with 30 second cooling intervals) followed by centrifugation at 1000*g* for 5 min. PSD activity of the extracts (in various amounts ranging from 50 to 437 µg) was measured using 0.2 mM phosphatidyl[1′‐^14^C]serine (400 cpm nmol^−1^) as the substrate, and the reaction product was trapped as ^14^CO_2_ on 2 M KOH‐impregnated filter paper, as described previously (Trotter *et al*., 1993). No detectable enzyme activities were found in *T. brucei* cell extracts with this method.

## Supporting information

Supporting Information
