# Supplementary material for: Phosphatidylserine synthase 2 and phosphatidylserine decarboxylase are essential for aminophospholipid synthesis in T rypanosoma brucei
Source: Mol Microbiol. 2017 Mar 2;104(3):412–27. doi: 10.1111/mmi.13637 (PMC5413845; doi:10.1111/mmi.13637)
Supplement: Supplementary file 1 — Supporting Information [file MMI-104-412-s001.pdf]

## Supplementary information

Figure S1

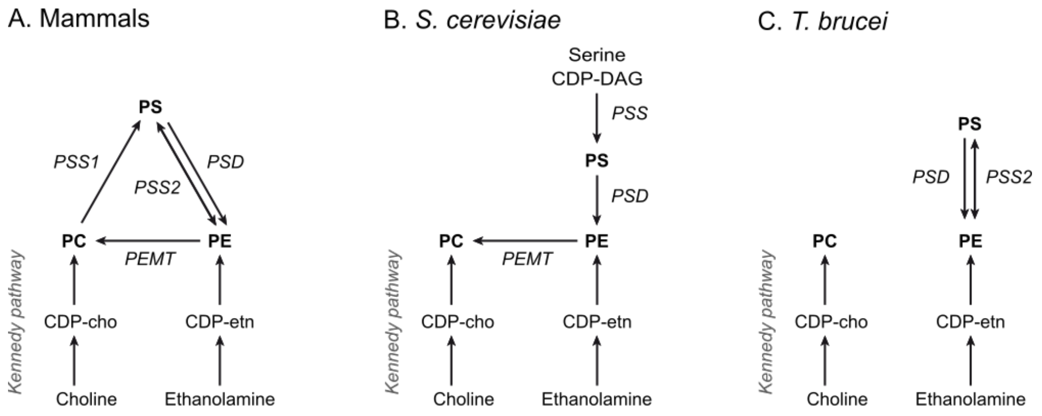


Figure S1. Comparative schemes of biosynthetic connections between PC, PE and PS in A. mammals, B. *Saccharomyces cerevisiae* and C. *Trypanosoma brucei*. The enzymes responsible for the reactions are indicated in italic: *PCS,* PC synthase, *PEMT;* PE methyltransferase; *PSD,* PS decarboxylase; *PSS1/2,* PS synthase 1 or 2; the notation *Kennedy pathway* includes ethanolamine/choline kinases, ethanolamine/choline cytidylyltransferases and choline/ethanolamine phosphotransferases.

Figure S2

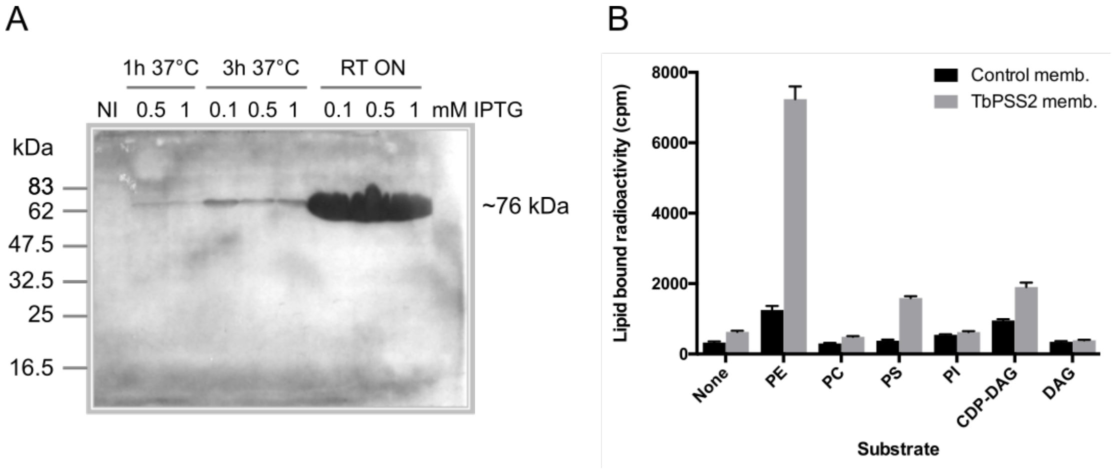


Figure S2. A. TbPSS2 was sub-cloned into pET32b expression vector and expression was induced for 1 or 3 hours at 37°C or at room temperature overnight (RT ON) with increasing concentrations of IPTG. Molecular mass markers are indicated in the left margin. B. Microsomal membranes from *E. coli* before (black bars) and after (grey bars) induction of TbPSS2 were isolated and PSS activity was assessed using [$^3$H]serine as substrate in the presence of various lipids. The values represent [$^3$H]-labeled lipids present in the organic phase after two-phase extraction. PE, phosphatidylethanolamine; PC, phosphatidylcholine; PS, phosphatidylserine; PI, phosphatidylinositol; CDP-DAG, CDP-diacylglycerol; DAG, diacylglycerol.

Figure S3

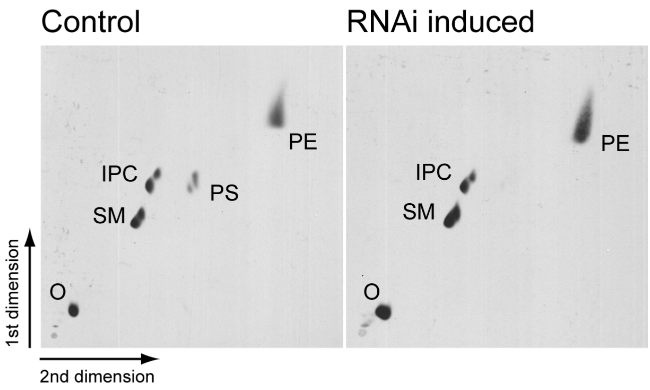


Figure S3. Lipid analysis of control and TbPSS2-depleted (RNAi induced) trypanosomes. Parasites were labeled overnight with [$^{3}$H]serine and phospholipids were extracted and separated by two-dimensional TLC. Plates were exposed to BioMax MS films and radioactive lipids were detected by fluorography. O, origin (site of sample application); SM, sphingomyelin; IPC, inositol phosphorylceramide; PS, phosphatidylserine; PE, phosphatidylethanolamine.

Figure S4

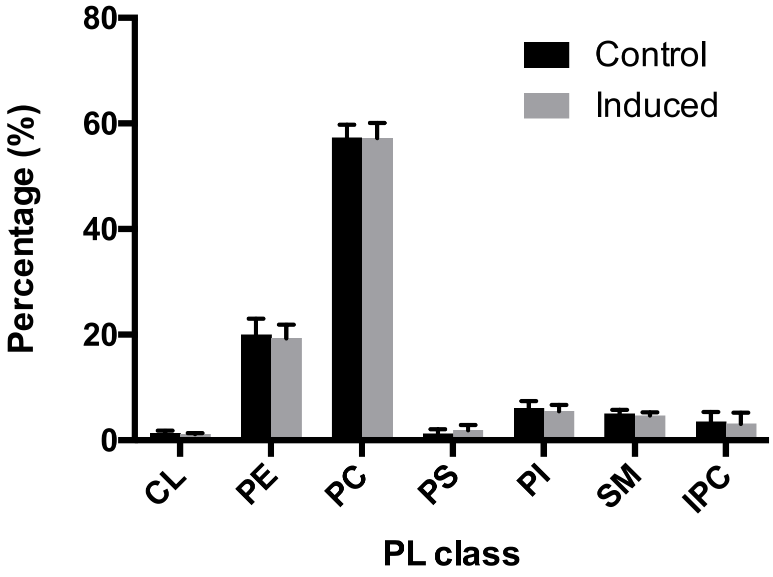


Figure S4. Phospholipid analysis of control and TbPSD-depleted (RNAi induced) trypanosomes. Parasites were cultured for 6 days in the absence (black bars) or presence (grey bars) of tetracycline to induce down-regulation of TbPSD. Subsequently, lipids were extracted, separated by two-dimensional thin layer chromatography and quantified by phosphorus analysis. The values represent means ± standard deviations from three independent experiments. CL, cardiolipin; PE, phosphatidylethanolamine; PC, phosphatidylcholine; PS, phosphatidylserine; PI, phosphatidylinositol; SM, sphingomyelin; IPC, inositol phosphorylceramide; PL phospholipid.

Figure S5

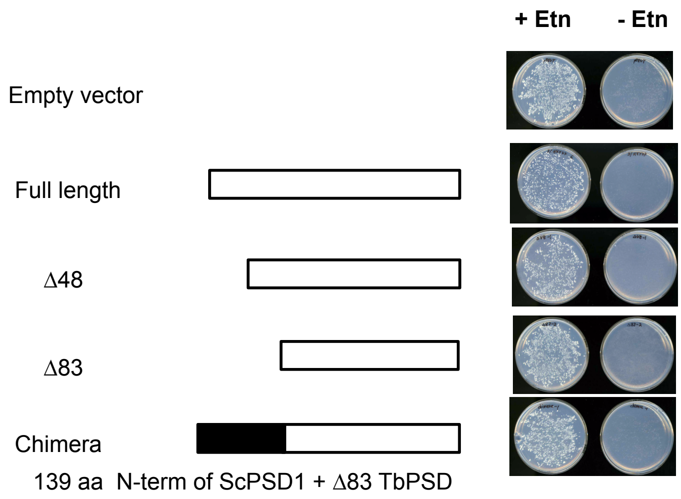


Figure S5. The TbPSD cDNA fails to complement the ethanolamine (Etn) auxotrophy of the *psd1Δpsd2Δdpl1Δ* strain. An empty vector (pBEVY), pBEVY-TbPSD, pBEVY-*Δ48*TbPSD, pBEVY-*Δ83*TbPSD, and pBEVY-chimera *Δ83*TbPSD were transformed into the *psd1Δpsd2Δdpl1Δ* strain. The transformants were selectively grown on uracil dropout synthetic glucose (SC-U) plates supplemented with 2 mM ethanolamine. The transformants were replica plated into the SC-U plates supplemented with or without ethanolamine (+/- Etn).

Figure S6

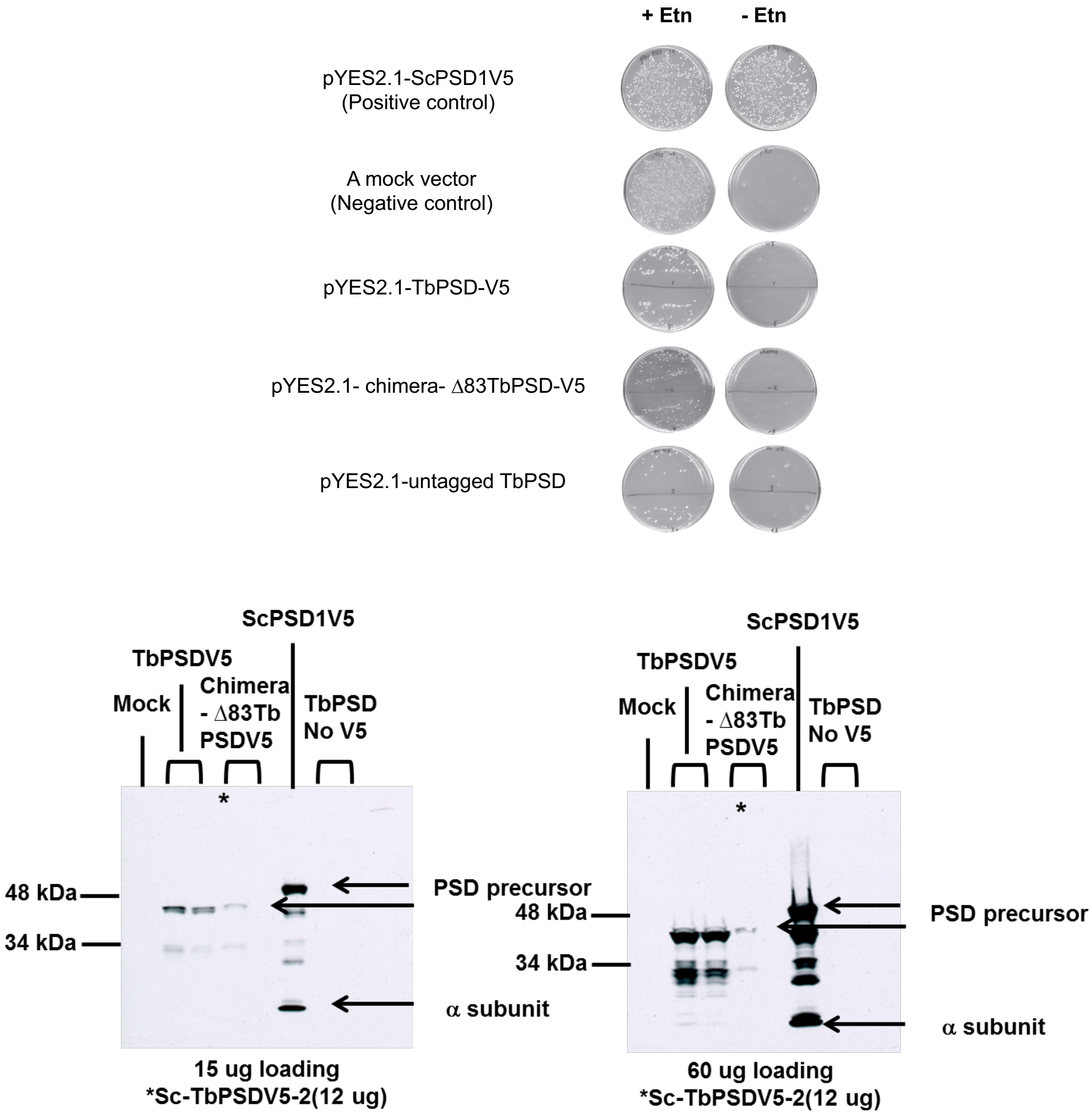


Figure S6. Expression of TbPSD-V5 fusion proteins in the *psd1Δpsd2Δdpl1Δ* strain. Top panel. *pYES2.1-ScPSD1-V5,* an empty vector, *pYES2.1-TbPSD-V5* or *pYES2.1-chimera Δ83TbPSD-V5,* or *pYES2.1 untagged TbPSD* were transformed into the *psd1Δpsd2Δdpl1Δ* strain. The transformants were selectively grown on uracil dropout synthetic galactose (SG-U) plates supplemented with 2 mM ethanolamine. The transformants were replica plated into the SG-U plates supplemented with or without ethanolamine (+/- Etn). Bottom panel. Immunoblot analysis was performed to detect the V5 epitope in proteins extracted from the *psd1Δpsd2Δdpl1*Δ mutant strains harboring a mock vector, *pYES2.1-TbPSD-V5*, or *pYES2.1-chimera Δ83TbPSD-V5 or pYES2.1-ScPSD1-V5,* or *pYES2.1 untagged TbPSD* using anti-V5 antibody.
